# Single center experience of endovascular abdominal aortic aneurysms repair

**DOI:** 10.1186/1749-8090-8-S1-O7

**Published:** 2013-09-11

**Authors:** R Akchurin, T Imaev, P Lepilin, A Kolegaev, A Komlev, I Pokidkin

**Affiliations:** 1 Cardiovascular Surgery, Russian Cardiology Research Center Moscow, Russian Federation

## Background

To present own results of endovascular repair of abdominal aortic aneurysms (EVAR AAA) using different techniques in patients with challenging anatomy and a wide range of different comorbidities being therefore unfit for open surgery due to high perioperative risk value.

## Methods

Since May 2010 51 patients with a median age of 69 years were included. Among them there were 32 (89%) men and 4 (11%) women. 28 (78%) patients had infrarenal localization of abdominal aortic aneurysms, 8 (22%) patients had juxtarenal localization of AAA. In 34 patients (94%) abdominal aortic aneurysms were etiologicaly atherosclerotic and in 2 patients (6%) it was Marfan syndrome to be the definite reason for aneurysmatic disease.

The maximum dimensions of the abdominal aorta averaged 59 ± 18mm. All patients had challenging anatomy: short and severely angulated and/or conic proximal aortic neck, unsuitable distal ”landing zone”, thrombus, calcification and complicated morphology, complex aneurysms and dissections of iliac and femoral arteries.

## Results

All patients got epidural/spinal anesthesia. 4 patients with juxtrarenal AAA were operated on with fenestrated endoprostheses and in 4 patients- “chimney” technique was used (including 1 patient with “sandwich” technique). In cases of complicated morphology of iliac arteries subclavian/axillary access was used. Also we made femoro-femoral or femoro-popliteal bypass in 3 patients with need of lower extremities revascularizaton. Still 2 patients revealed acute in-stent thrombosis due to dissection of distal part of external iliac artery and due to crushed chimney-stent successfully managed with balloon angioplasty and supplementary stent implantation. Hospital mortality was 2,8% (1 patient died due to acute MI on 5 day after procedure). 30-day’s mortality was 2,8% as well.

## Conclusion

EVAR is the promising alternative to conventional open aortic repair.

